# Cervical-thoracic facial necrotizing fasciitis of odontogenic origin

**DOI:** 10.1590/S1808-86942011000600019

**Published:** 2015-10-19

**Authors:** Rui Medeiros Júnior, Auremir da Rocha Melo, Hugo Franklin Lima de Oliveira, Silvana Maria Orestes Cardoso, Carlos Augusto Pereira do Lago

**Affiliations:** 1 Oral and maxillofacial surgery and trauma specialist, Hospital da Restauração, Recife, PE. Oral and maxillofacial surgeon; 2 Oral and maxillofacial surgery and trauma specialist, Hospital da Restauração, Recife, PE. Oral and maxillofacial surgeon; 3 Medical resident in oral and maxillofacial surgery, Hospital da Restauração, Recife, PE. Dental surgeon; 4 Doctoral degree in oral biology, Paris VII University, France. Professor of the Orofacial Surgery and Prosthesis Department, Pernambuco Federal University, UFPE, Recife, PE; 5 Doctoral de gree in oral and maxillofacial surgery and trauma, Pernambuco Dentistry School (FOP), UPE. Staff of the Oral and Maxillofacial Surgery and Trauma Unit, Hospital da Restauração, Recife, PE. Restauracao Hospital (Hospital da Restauração)

**Keywords:** face, fasciitis, necrotizing, infection

## INTRODUCTION

Necrotizing fasciitis (NF) of the head and neck is a rare and potentially fatal soft tissue bacterial infection that affects mostly male and female adult and elderly patients.^1^ There are no reliable data on its true incidence in the population.^2^

The origin is odontogenic in most cases, resulting from dental abscesses, chronic periodontal disease, or pharyngeal diseases. It progresses by forming extensive necrosis and gas in subcutaneous tissues and the underlying fascia, and has a high mortality rate (about 40%).^2^

This disease is usually polymicrobial; such cases may be classified into type I, when caused by a mixed flora consisting of obligate anaerobic bacteria and other facultative anaerobic organisms not belonging to group A, and type II, when group A *Streptococcus* singly or with *Staphylococcus aureus* is involved.^3^

Risk factors for NF are uncontrolled diabetes mellitus, peripheral vascular disease, liver diseases, and immune diseases.^2^ Imaging is essential to define the topography of the infection; the differential diagnosis is made mostly with cellulitis and initial stage erysipela.^4^ Successful treatment requires an early diagnosis, radical surgical debridement of all necrotic tissues, endovenous broad-spectrum antibiotic therapy, and aggressive general support measures.^4^

## CASE REPORT

A male patient aged 37 years with a history of chronic alcohol abuse presented a facial-cervical-thoracic lesion that suggested cellulitis, and a history of untreated dental infection.

**Figure 1:**
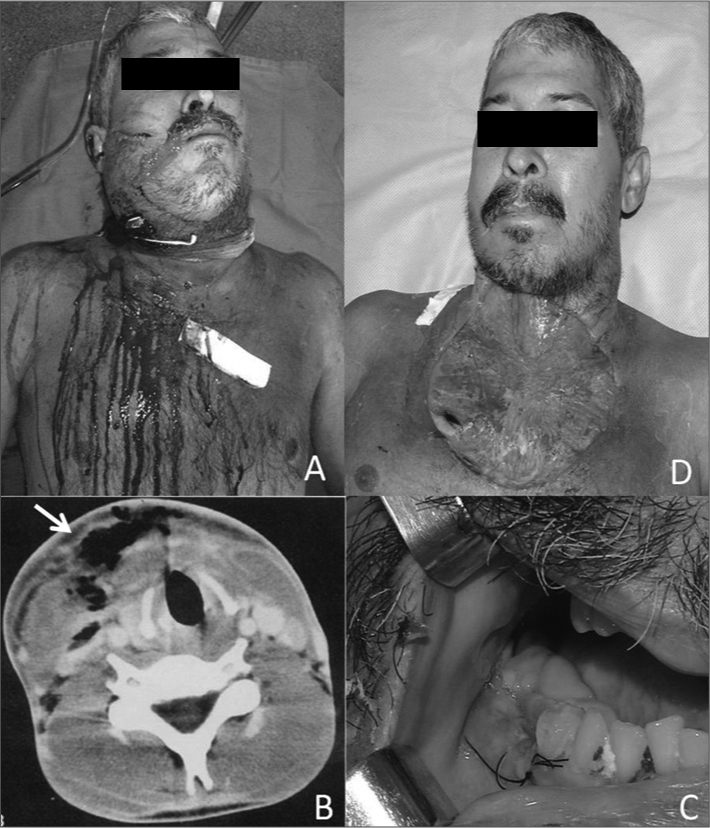
A) Extensive erythematous area associated with bilateral submandibular, submentonian, and sublingual edema extending to the neck and thorax. B) Subcutaneous emphysema of the neck - computed tomography. C) Removal of many teeth roots in the mandible (primary infection sites). D) The surgical wound on the sixth post-operative day. Note the extensive open area and residual cavity with pus.

On the physical examination, there was mandibular trismus, the submandibular, sublingual, and submentonian regions were enlarged, painful, and hardened bilaterally; this extended to the thorax (Fig. 1A). An oral examination showed several remaining tooth roots, teeth with caries, and periodontal disease. Hyperemia, hyperthermia, tachypnea, dehydration, and leukocytosis indicated sepsis. Computed tomography revealed a characteristic subcutaneous emphysema (Fig. 1B).

Emergency treatment was started, involving radical surgical debridement. During surgery, material was taken and sent for culture and an antibiogram. The anterior neck and thoracic muscles were left exposed. Multiple removal of teeth were done to remove the primary disease (Fig. 1C).

Antibiotic therapy was started empirically with ceftriaxone and metronidazole. The culture revealed *Klebsiella pneumoniae* and antibiotic sensitivity testing showed that it was sensitive to the aforementioned antibiotics. Additional smaller areas were debrided during the first two weeks after surgery (Fig. 1D).

## DISCUSSION

The present study presents a case of NF of odontogenic origin in a patient with chronic alcohol abuse, which progressed rapidly to the neck and thorax. Whitesides et al.^5^ reported that 81% of such cases start from the second or third inferior molars. Kaul et al.^6^ studied 77 cases and found at least one underlying disease in over 70% of them.

The diagnosis of NF is essentially medical (based on non-elastic skin edema, hypoesthesia, subcutaneous crepitus, etc.). It is confirmed during surgery by noting poor adherence of subcutaneous tissues, lack of bleeding, and necrosis of the fascia.

Emergency surgery was done after computed tomography showed a characteristic subcutaneous emphysema and revealed the true extent of the infection. Aggressive debridement was done of the neck and thorax; the procedure reached the mammillary line, the midpoint of the clavicle bilaterally, and the base of the mandible. The findings at surgery included a decreased resistance to dissection, necrosis in deeper layers of the fascia, necrosis of the skin and subcutaneous tissue, drainage of pus, and a fetid odor, which sealed the diagnosis of NF.

A rare finding was *Klebsiella pneumoniae* as a single causative bacteria, which is not typical of the classic polymicrobial pattern of infection. Antibiotic therapy was effective in this case. Adjuvant measures, such as immunoglobulins and hyperbaric oxygen therapy, are also currently employed.^3^

## FINAL COMMENTS

Rarely oral infection may cause severe and even fatal NF. This condition progresses rapidly, especially when associated with predisposing factors. The treatment requires a prompt diagnosis, broad-spectrum antibiotic therapy, and radical surgery. A multidisciplinary team is recommended in many cases.
